# Clinical efficacy of different open approaches in the surgical treatment of thoracolumbar tuberculosis: a single-center retrospective comparative study

**DOI:** 10.1186/s13018-023-03834-1

**Published:** 2023-05-12

**Authors:** Tianji Wang, Zhensheng Ma, Wei Lei, Zixiang Wu, Huifa Xu, Tiancheng Ma, Tianqing Li

**Affiliations:** Department of Orthopedics, Xijing Hospital, The Fourth Military Medical University, Xi’an, 710032 China

**Keywords:** Thoracolumbar tuberculosis, Surgical approach, Outcomes, Complications

## Abstract

**Objective:**

To assess the clinical efficacy of three different surgical approaches in the treatment of thoracolumbar tuberculosis.

**Methods:**

A total of 138 patients with thoracolumbar tuberculosis treated by open surgery were retrospectively analyzed. The surgical methods were divided into anterior, posterior and anterior–posterior combined. The hospital stays, amount of bleeding, operative time, preoperative, postoperative and last follow-up ESR, CRP, Frankel score, ODI, VAS, correction and loss rate of kyphosis, fusion rate and complications were recorded and analyzed.

**Results:**

The average follow-up was 66 months. The average hospital stay, operative time and amount of bleeding of the anterior–posterior combined group were higher than other groups (*P* < 0.05). ESR and CRP of all patients were reduced postoperatively (*P* < 0.05). No significant difference among the three groups was found in the postoperative correction angle of kyphosis (*P* < 0.05), while the pre- and postoperative Cobb angle as well as correction rate had significant differences. The posterior approach could achieve better correction, and the loss of correction was more in the anterior group, 40.9 percent of patients performed correction loss. The Frankel score, VAS and ODI were significantly reduced among the three groups, and the incidence rate of complications of the anterior approach was lower than the other groups, with a significant difference (*P* < 0.05).

**Conclusion:**

The anterior approach has more advantages and fewer complications, which is supposed to give preference to and could not be replaced by the posterior and anterior–posterior combined approach.

## Introduction

Spinal tuberculosis, the most type of musculoskeletal tuberculosis, occurs commonly as an extremely severe and dangerous infectious disease in the world and accounts for 50–60 percent of bone and joint tuberculosis [1]. Surgery is a common method for the treatment of spinal tuberculosis. Hodgson et al. and Fang et al. indicated that the anterior spinal tuberculosis lesion removal combined with bone grafting fusion was available, while Konstam et al. suggested chemotherapy alone [2]. Konstam and Blesovskky reported 207 cases of spinal tuberculosis who received simple chemotherapy treatment and achieved an 86% cure rate [3]. However, the better therapeutic regimen for spinal tuberculosis treatment was controversial. Scholars from Asia and Africa conducted a series of multi-center randomized controlled studies and compared multiple research topics including the surgical treatment and nonsurgical treatment, different chemotherapy regimens, simple lesion removal and bone graft fusion; they indicated that the long-term cure rate was not significantly improved by surgical treatment, compared with drug therapy alone for patients with mild vertebral destruction, less abscess and light inflammatory response [4–7]. In the last two decades, with the development of the advances in spinal surgery and the invention of internal fixation, the internal fixation instrument has been widely applied in the treatment of spinal tuberculosis due to the defects of traditional surgical method such as bone graft shedding and absorption. Traditional open surgery is beneficial to clinical outcomes through lesion removal, nerve decompression, kyphosis correction and reconstruction of spinal stability [8]. Surgical approaches include anterior, posterior and anterior–posterior joint approaches [9]. All three methods could achieve good healing and long-term clinical outcomes reported by previous studies [10–12]. The anterior approach was reported with thorough debridement and relatively convenient bone grafting, which can better restore the height of the vertebral body, while the wound is large [13]. The posterior approach could achieve debridement and bone graft by a surgical incision while breaching the spinal stability and possibly transferring the focus to the rear [14, 15]. The disadvantages of the anterior–posterior joint approach include operation trauma, long anesthesia time, frequent position change and a wide range of complications [9].

**Fig. 1:**
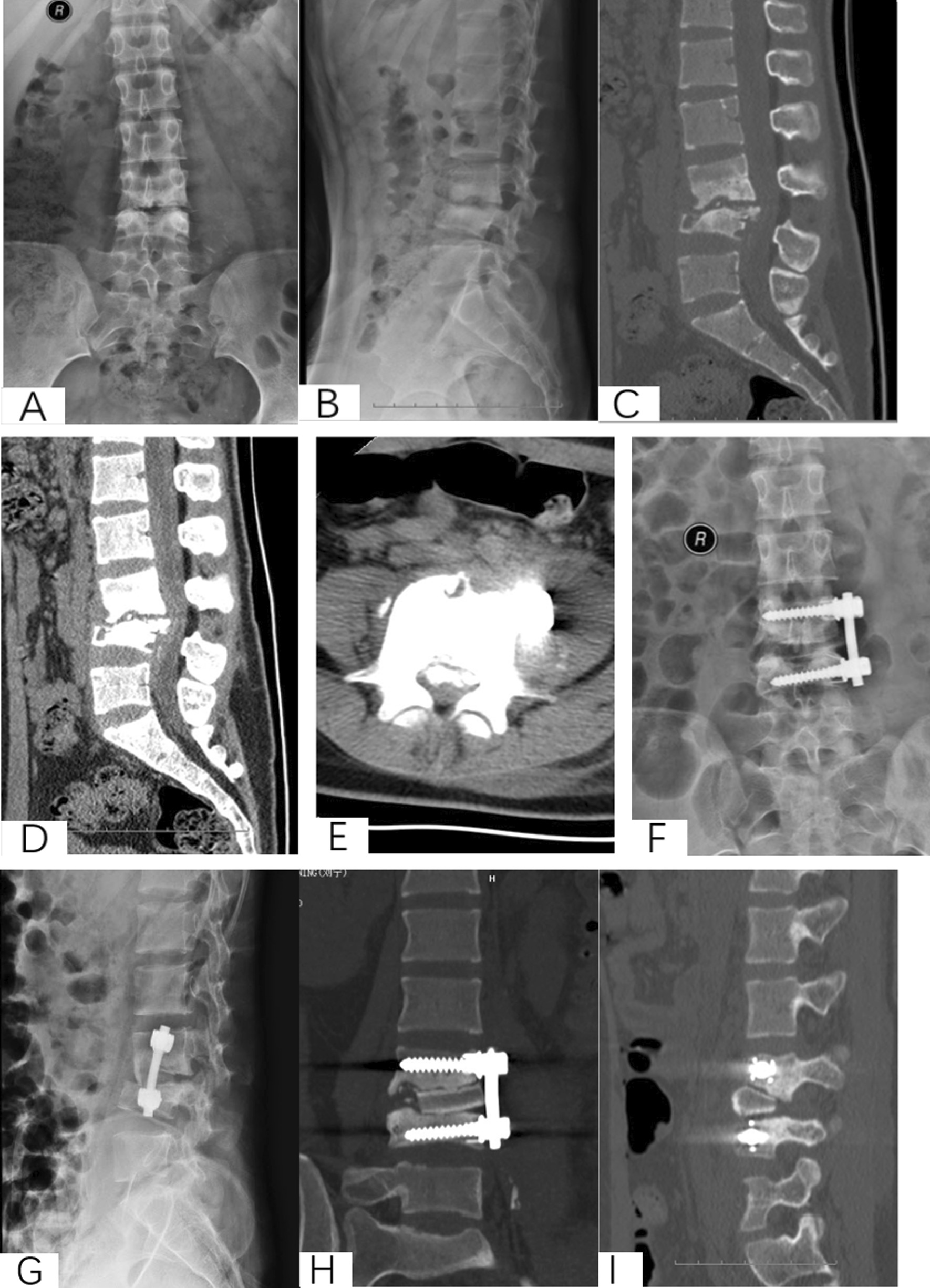

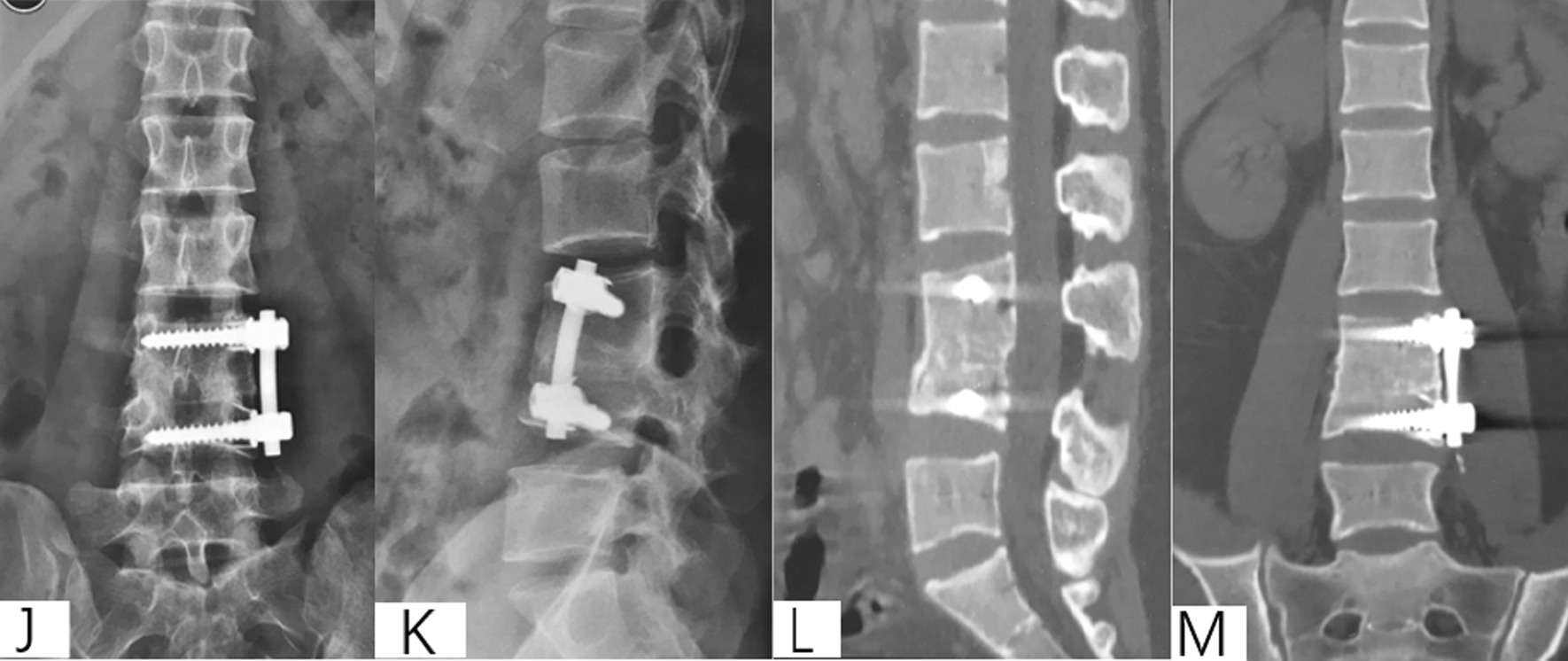
An 18-year-old female with L3-4 STB in anterior approach group. **A**–**E** Preoperative X-ray and CT showed that the vertebral tuberculosis and paravertebral abscess were formed. **F–I** Postoperative X-ray and CT. **J**–**M** X-ray and CT at 6 years postoperative showed good bone healing

Nevertheless, it is still inconclusive which approach for open surgery to treat thoracolumbar tuberculosis should be chosen. The anterior approach was mostly advocated for reliable efficacy. However, the indication and outcomes of different surgical approaches are still ambiguous. In this study, three approaches were applied to treat thoracolumbar tuberculosis, to provide an evidence-based comparison for the clinical choice of surgical treatment (Fig. 1).

## Methods

**Fig. 2:**
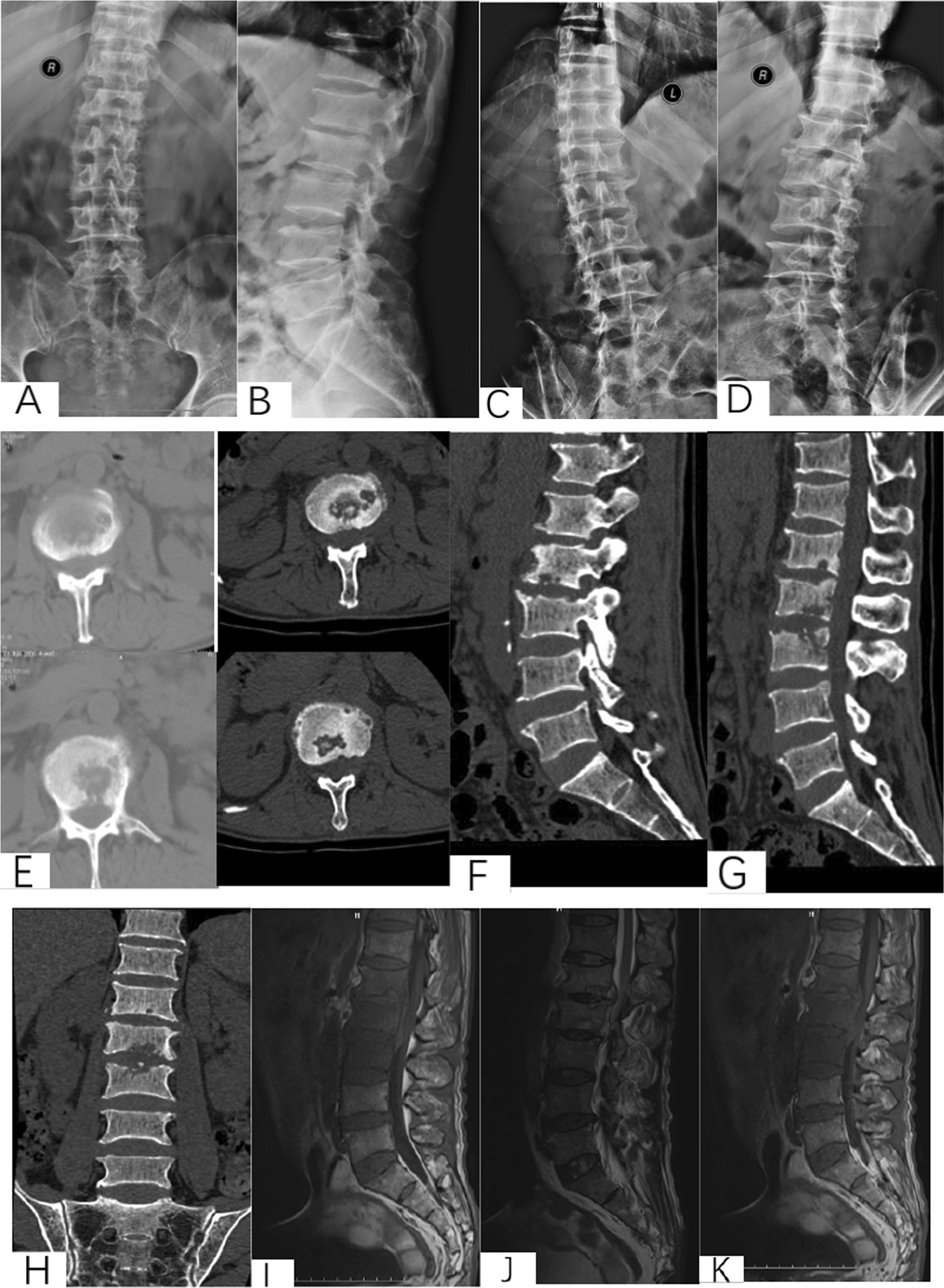

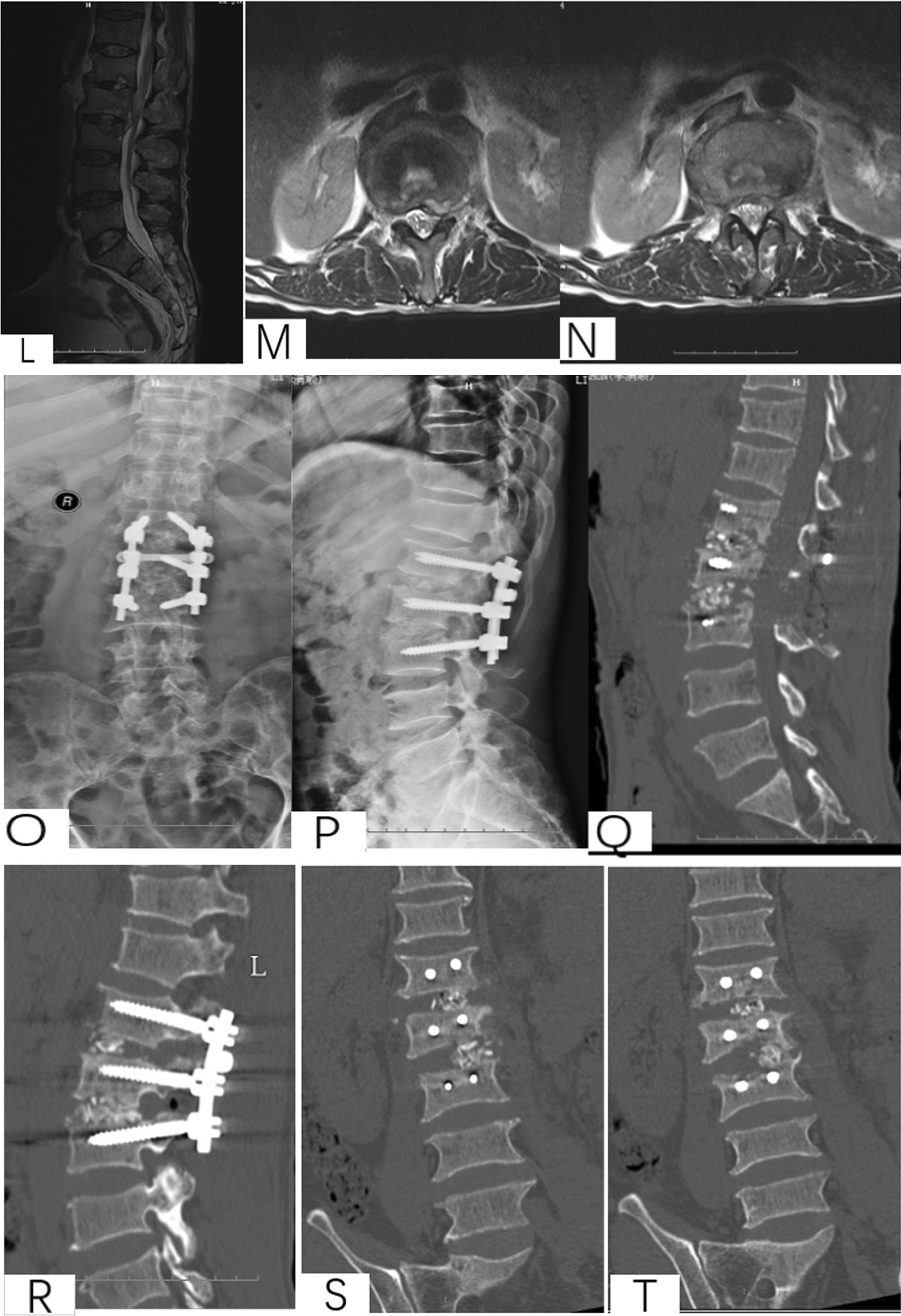

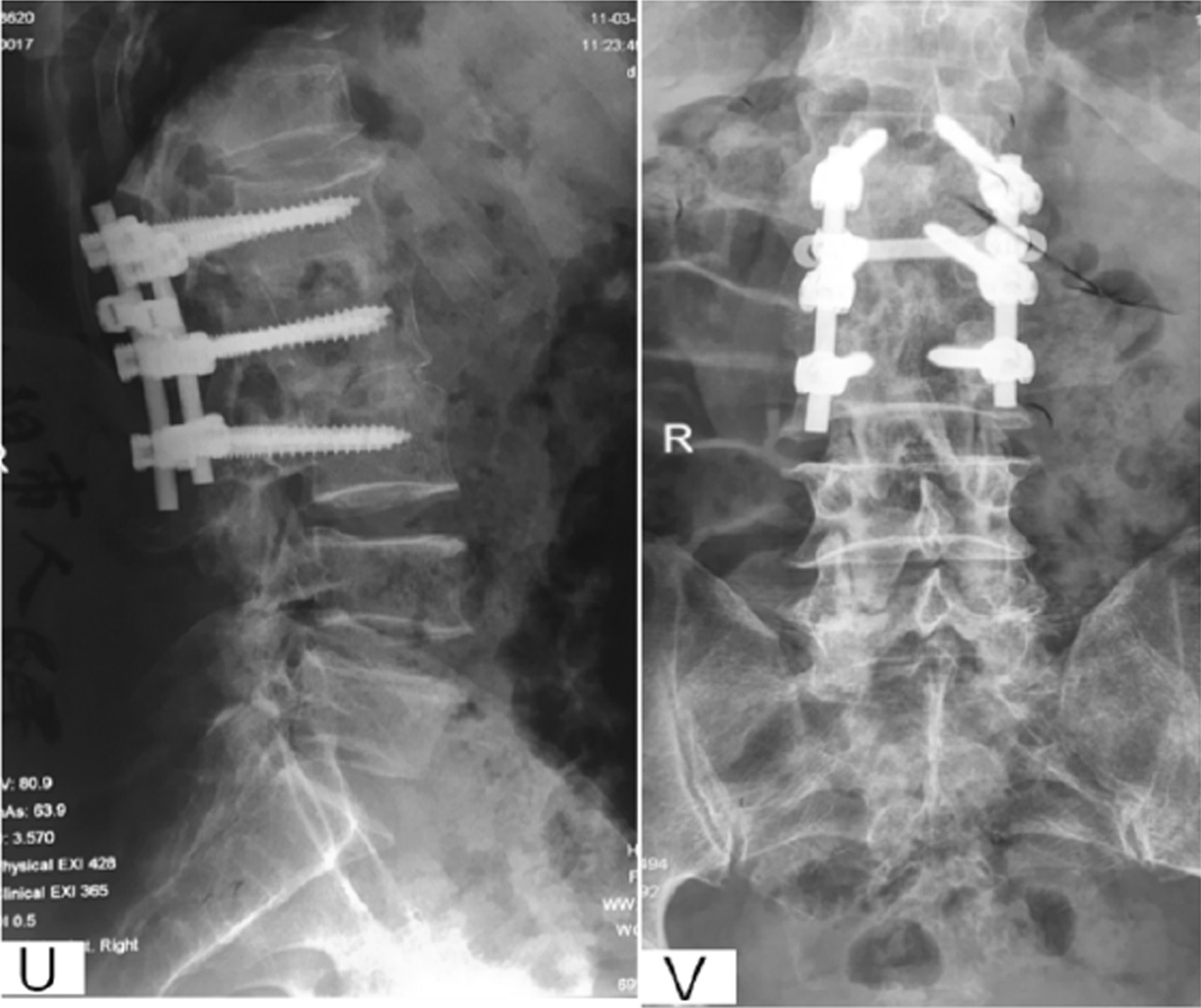
A 57-year-old male with L1-3 STB in posterior approach group. **A**–**M** Preoperative X-ray, CT and MRI showed the vertebral tuberculosis. **O**–**T** Postoperative X-ray and CT. **U**–**V** X-ray at 5 years postoperative showed good bone healing

Patient selection. This investigation was conducted as a retrospective observational cohort study at Xijing Hospital of the Fourth Military Medical University from 2009 to 2017 under retrospective guidelines of the hospital. In total, the medical records of 138 patients diagnosed with thoracolumbar tuberculosis who underwent surgery were analyzed. According to the surgical approaches, patients were divided into anterior surgery, posterior surgery and anterior–posterior surgery groups. The scopes of surgery for all patients were no more than one intervertebral space. All surgeries were carried out by two same experienced surgeons from the Orthopedics Department of Xijing Hospital (Fig. 2).

**Fig. 3:**
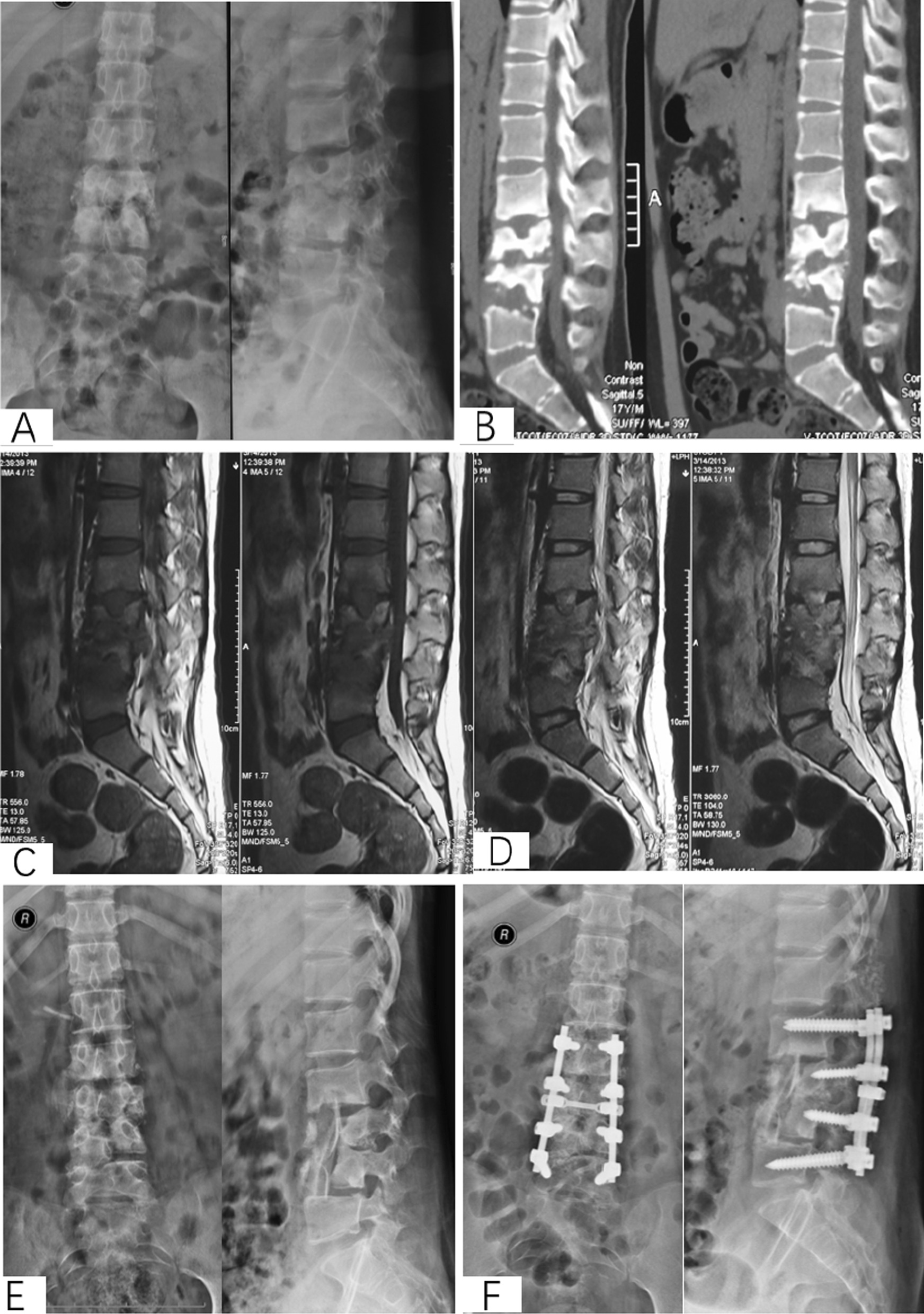

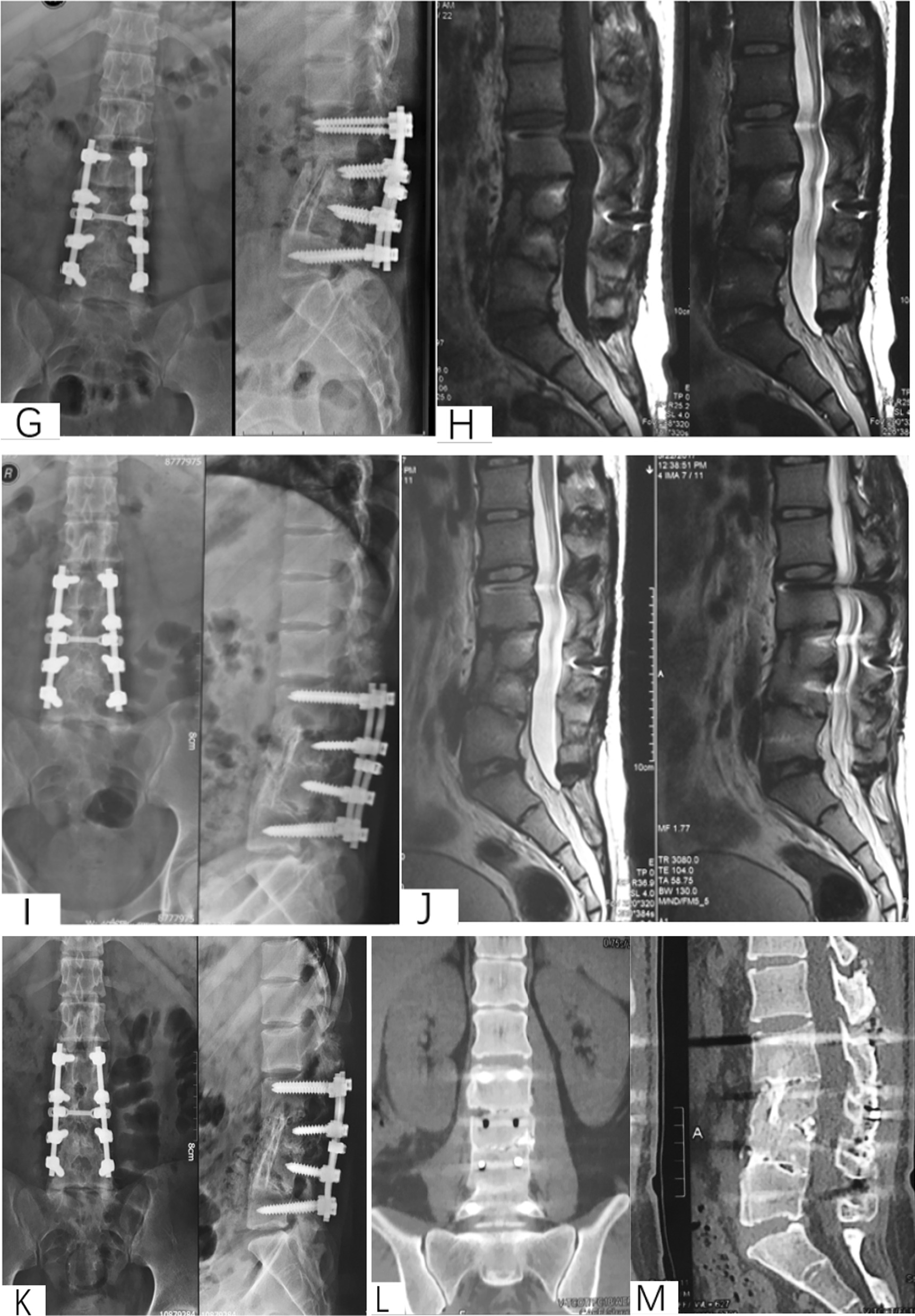
A 16-year-old male with L2-4 STB in anterior–posterior approach group. **A–D** Preoperative X-ray, CT and MRI showed the vertebral tuberculosis and paravertebral abscess. **E** Postoperative X-ray of anterior approach. **F** Postoperative X-ray of posterior approach. **G**–**H** X-ray and MRI at 1 year postoperative. (**I**–**J**) X-ray and MRI at 4 years postoperative. **K**–**M** X-ray and CT at 5 years postoperative

Preoperative management. All patients underwent X-ray, CT and MRI and routine surgical examination preoperatively. Besides, patients were treated with regular ant-TB chemotherapy 4–6 weeks before surgery (isoniazid 0.3 g/d, rifampicin 0.6 g/d, pyrazinamide 0.75 g/d and ethambutol 0.75 g/d) (Fig. 3).

Surgical methods. ①Anterior approach: Anterior surgery for thoracic vertebral tuberculosis generally adopts intrathoracic and extrapleural debridement and transthoracic debridement. (a) Intrathoracic and extrapleural debridement: the patient is placed in a lateral recumbent position, with the serious lesion on the upper side. The incision starts at the outer border of the paraspinal muscles and extends along the ribs to the midclavicular line. For thoracic 4–5 vertebral tuberculosis, the incision is made between the medial side of the scapula and the spinous process, at the plane of the second rib, and goes down along the medial border of the scapula, bypassing the subscapular angle and ending at the anterior axillary line of the lateral chest wall. Debridement of lesions and internal fixation with bone grafting: Cut the outer muscle, expose the ribs, peel off the periosteum, cut off the ribs for bone grafting, and carefully and bluntly peel off the pleura, the medial side should reach the midline of the spine, and if the paravertebral abscess is relatively large, it should be enlarged Peel range. Use a distractor to retract the chest wall to reveal the abscess, puncture and locate the abscess to reach the diseased vertebra, thoroughly clean the lesion, decompress the spinal canal with intraspinal compression, chisel out the bone graft bed, and use filling or supporting graft according to the size of the defect area. Bone and bone graft materials are made of three-sided cortical iliac bone and autologous rib. Gelatin sponge is placed in the lesion to stop bleeding, and streptomycin powder is sprayed. If internal fixation is installed, when the residual vertebral body exceeds half of the lesion after cleaning, the vertebra is placed in the lesion. Body nails, otherwise extend the fixing gap, generally no more than three gaps, and use nail rods or nail plates to fix. (b) For transthoracic debridement (T1-T4), double-lumen intubation can be used for general anesthesia to shrink the lungs on the operative side, so as to facilitate intraoperative exposure of the lesions. The right-side approach is adopted, which avoids the common carotid artery and subclavian artery in the upper part of the left mediastinum. The patient is placed in the left lateral decubitus position with the right thoracic cavity above the incision. Cartilage. Lesion debridement and bone grafting: incision of the muscle, retraction of the scapula to one side, excision of part of the rib, alternate blunt and sharp separation when thoracic adhesions, separation of the lung layer from the parietal layer, proper hemostasis and exposure of the natural lung after the thoracic cavity. Shrink. The pleura was incised and separated from both sides, hemostasis, puncture to locate the abscess, thorough cleaning of the lesion. The following steps are the same as above. (c) Transthoracic debridement (T4-T12), general anesthesia, double-lumen intubation, atrophy of the operative side of the lung, in order to facilitate intraoperative exposure of the lesion. The patient is in a lateral decubitus position with the severely affected side up. The incision starts from the medial side of the scapula and between the spinous processes and the plane of the second rib, goes down along the medial border of the scapula, bypasses the subscapular angle, ends at the anterior axillary line of the lateral chest wall, incises the muscle, removes part of the rib, stops the bleeding and separates bluntly and sharply alternately. Separate the lung layer from the parietal layer, properly stop the bleeding, and pull the lung to the midline with a wide retractor. For bronchial intubation, the lung on the surgical side can shrink, and the prevertebral abscess can be touched under direct vision. The chest wall was retracted with a distractor to reveal the abscess, and the abscess was punctured and positioned fluoroscopy. When the abscess is large, the artery may be surrounded by the abscess, and it is necessary to avoid clamping or ligating the artery near the aorta or intervertebral foramen. Through the abscess to reach the diseased vertebra, thoroughly clean the lesion. The following steps are the same as above. (d) Debridement of lesions through the extraperitoneal approach (L1-S1), lateral recumbent position, slightly backward, and put a cushion under the armpit to protect the axillary nerve. Oblique incision, the starting and ending points of the incision are adjusted according to the height of the lesion. Exposure: The external oblique, internal oblique and transversus abdominis are the main muscles encountered in the anterolateral approach, and the external oblique, internal oblique and transversus abdominis are incised sequentially. After the three-layer abdominal muscle was incised, the abdominal aorta or inferior vena cava was protected and pushed open with wet gauze, the abscess was explored, and the psoas major muscle was bluntly separated. Puncture the abscess for localization. The saline-wet gauze protects the surrounding tissues and cuts the abscess. When the abscess is large, the arteries and veins may be surrounded by the abscess and reach the diseased vertebra through the abscess. The lesions are thoroughly cleaned. The following steps are the same as above. ②Posterior approach: Posterior debridement and bone grafting internal fixation, prone position under combined epidural anesthesia or tracheal intubation, thoracic and lumbar tuberculosis are often performed with posterior median approach for laminar decompression, lesion removal and bone grafting fusion and internal fixation; usually the body surface markers for the posterior approach of the thoracolumbar spine include the lumbar spinous process, the line connecting the iliac spine and the lumbar 3 transverse process, etc. The diseased segment is identified in advance. The anti-pressure sticker is attached to the bony protrusion to prevent skin pressure injury, and the convenience of intraoperative fluoroscopic filming is also considered. Conventional incision in the posterior median, muscle stripping and conventional screw placement. The cephalic screws of the spine should be as close to the upper endplate as possible, and the caudal screws should be as close to the lower endplate as possible to keep the internal fixation screws away from the lesion area. When the sacral tuberculosis is severely damaged, it is necessary to extend the fixation segment. From the segment to the ilium, pay attention to protect the nerve root. Abscesses or tuberculosis granulomas that enter the spinal canal can be seen. Clean up the lesion, properly spread the lesion, measure the size of the lesion and the location of the defect. When the defect is small, the ilium and the autogenous normal spine are taken. The laminae are mixed with streptomycin powder and filled, and compacted with instruments. When the defect is large, a large piece of iliac bone or autologous bone can be implanted into the intervertebral body, and then, the remaining bone can be filled in the intervertebral space and compacted. After checking that the nerve is not compressed or abnormal, the internal fixation is locked and the wound is closed. ③Anterior–posterior approach: Anterior debridement, posterior internal fixation, anterior debridement, bone graft fusion and posterior internal fixation were completed in two incisions in one or two stages, respectively. Transthoracic surgery was performed under general anesthesia with intubation or double-lumen bronchial intubation. Tube. The patient is placed in the lateral recumbent position, with the severely affected side on the upper side. Transthoracic or anterior transabdominal surgery is the same as the anterior surgical procedure. About 1 week after the operation, the patient is placed in the prone position, and the posterior median incision is made to expose the lamina and the outer edge of the articular process. One or two segments above and below the lesioned vertebral body are fixed and fused, and the proximal screw of the lesion destroys the vertebral body as close as possible to the upper endplate of the vertebral body or the lower endplate of the vertebral body, so that the internal fixation screw is far away from the lesion area. Short pedicle screws, laminar surface and facet joint space and posterolateral bone grafting were performed at the same time, and autologous iliac bone was selected.

Postoperative management. After surgery, patients were asked to stay in bed and get proper functional exercise to prevent deep vein thrombosis of the lower extremity. Incision drainage was removed 48–72 h after the operation when the volume was less than 50 ml/d. All patients were requested to continue anti-TB chemotherapy for 12–18 months. During this time, erythrocyte sedimentation rate (ESR), C-reactive protein (CRP), hepatorenal function, vital signs and motion sensation of lower limbs were detected. The brace was applied for postoperative 3–6 months. Besides, X-ray, ESR, CRP and hepatorenal function were followed up 1, 3, 6 months postoperatively. Frankel scores, Oswestry Disability Index (ODI) and visual analogue scale (VAS) were inquired, and the Cobb angle, the fusion rate and complications were collected.

Clinical outcome. The four degrees of the clinical cure classification include ‘Outstanding, Good, Ordinary and Bad.’ Outstanding: Symptoms of tuberculosis and pain in the lower back and legs disappeared, and the physical labor was fully recovered one year postoperatively. The lesions disappeared in X-ray, and the intervertebral bone fusion was formed without internal fixation loosening. Good: The patients performed occasionally mild pain and were able to engage in light physical labor after 1 year. X-ray showed no loosening of the internal fixation, while the deformity angle was lost 5–10°. The intervertebral bony fusion was insufficient, while no false joints existed. Ordinary: Postoperative tuberculosis symptoms persisted for 3 to 4 months, and X-rays showed incomplete intervertebral fusion after 1 year, and the patients could only engage in general physical labor. Bad: The incision healed by first intention after the operation, but the symptoms of tuberculosis lasted for 3 to 4 months. After 1 year, the patients could not do physical work except daily life. X-ray showed that the internal fixation loosening and the bone fusion was not good.

Statistical analysis. The data were analyzed with SPSS 20.0 Software. Independent t tests and chi-square tests were used to examine the preoperative, postoperative and last follow-up. The Kruskal–Wallis H test was analyzed to calculate the mean rank to assess the therapeutic efficacy of different approaches. Quantitative data were represented in mean ± standard deviation (SD) or percentage (%). *P* < 0.05 was considered as a significant difference.

## Results

A total of 138 patients were finally followed up, including 44 in the anterior group, 42 in the posterior group and 52 in the anterior–posterior group. No significant differences were found in gender (*P* = 0.954) and age (*P* = 0.761) among the three groups. The average follow-up time was 66 months (range from 16 to 108 months), with no significant difference (*P* = 0.149). All patients were cured clinically (Table 1).

**Table 1:** Comparison of general data of different open surgery approaches (*n* = 138, ‾x ± s)

	Anterior (*n* = 44)	Posterior (*n* = 42)	Anterior–Posterior (*n* = 52)	*P*
*Pre-op*				
Gender (m/f)	25/19	24/18	31/21	0.954
Age	45.00 ± 8.771	46.33 ± 8.035	45.63 ± 8.272	0.761
Follow-up	61.30 ± 15.734	70.29 ± 21.678	66.56 ± 24.911	0.149
ESR	52.45 ± 16.043	52.90 ± 14.922	52.67 ± 16.366	0.991
CPR	21.714 ± 7.044	22.381 ± 10.531	19.913 ± 10.797	0.436
ODI	12.34 ± 1.976	13.00 ± 1.848	12.94 ± 1.787	0.186
VAS	5.91 ± 1.137	6.00 ± 1.126	6.19 ± 1.205	0.473
Kyphosis angle	17.705 ± 7.902	17.714 ± 7.409	15.538 ± 6.188	0.228
Hospital stays	13.55 ± 1.970	13.40 ± 1.768	15.06 ± 2.940	0.001
Bleeding	295.45 ± 103.978	385.24 ± 66.744	482.69 ± 55.910	0.000
*Post-op*				
Operative time	140.30 ± 17.727	153.10 ± 18.707	209.13 ± 22.223	0.000
ESR	37.84 ± 11.658	29.38 ± 11.234	37.77 ± 11.676	0.001
CPR	10.520 ± 5.5484	10.807 ± 5.5023	11.492 ± 6.2294	0.699
ODI	9.27 ± 2.027	9.29 ± 2.075	9.46 ± 2.305	0.890
VAS	2.68 ± 0.561	2.60 ± 0.587	2.63 ± 0.561	0.779
Kyphosis angle	4.068 ± 2.585	1.857 ± 1.647	3.365 ± 2.629	0.000
Correction angle	13.636 ± 8.198	15.857 ± 7.621	12.173 ± 6.641	0.062
Correction rate (%)	71.34 ± 28.64	87.38 ± 13.36	74.47 ± 27.99	0.007
Correction angle loss	0.359 ± 0.564	0.405 ± 1.037	0.019 ± 0.431	0.000
*Last*				
Loss ratio (%)	40.90	19.05	11.50	0.002
ESR	5.02 ± 2.074	4.69 ± 2.030	2.063 ± 1.3637	0.768
CPR	1.889 ± 1.1614	1.860 ± 1.2214	4.87 ± 2.223	0.691
ODI	2.05 ± 1.642	2.12 ± 0.861	2.40 ± 1.672	0.445
VAS	0.20 ± 0.408	0.21 ± 0.415	0.27 ± 0.448	0.722
Kyphosis angle	4.427 ± 2.5677	1.667 ± 1.4595	3.346 ± 2.5582	0.000

The average hospitalization time, operation time and bleeding volume of patients in the anterior–posterior group were significantly higher than those in the other two groups (*P* < 0.05). ESR and CRP in the three groups were significantly lower than those before the operation (*P* < 0.05). At the last follow-up, ESR and CRP were both significantly reduced in three groups compared with those preoperative and postoperative (*P* < 0.05) (Table 2).

**Table 2:** Comparison of biochemical test between three surgery approaches before and after surgery (*n* = 138, ‾x ± s)

		Pre-op	Post-op	Last	*P*
Anterior	ESR	52.45 ± 16.043	37.84 ± 11.658	5.02 ± 2.074	< 0.001
*n* = 44	CRP	21.714 ± 7.0443	10.520 ± 5.5484	1.889 ± 1.1614	< 0.001
Posterior	ESR	52.90 ± 14.922	29.38 ± 11.234	4.69 ± 2.030	< 0.001
*n* = 42	CRP	22.381 ± 10.531	10.807 ± 5.5023	1.860 ± 1.2214	< 0.001
Anterior–posterior	ESR	52.67 ± 16.366	37.77 ± 11.676	4.87 ± 2.223	< 0.001
*n* = 52	CRP	19.913 ± 10.797	11.492 ± 6.2294	2.063 ± 1.3637	< 0.001

There was a significant difference between the preoperative and postoperative kyphosis angle of the three groups, while no significant difference in kyphosis angle was found in comparison among the three groups (*P* > 0.05). However, there was a significant difference in the kyphosis correction rate among the three groups. The effect of posterior surgery was better than anterior–posterior surgery, but there was no significant difference in the correction loss rate between anterior and anterior–posterior surgery. 40.9% of the anterior patients showed loss of kyphotic angle correction during follow-up (Table 1).

Frankel scores, VAS and ODI of the three groups in postoperative and last follow-up were significantly lower than those before the operation (*P* < 0.05) (Tables 1 and 3). There was no significant difference in Frankel score and clinical healing rate among the three surgical approaches (Table 4).

**Table 3:** Comparison of Frankel before and after surgery (*n* = 44, %)

	Frankel	Pre-op	Post-op	Last	*P*
Anterior *n* = 44	A	1 (2.3%)	1 (2.3%)	1 (2.3%)	0.048
	C	3 (6.8%)	0 (0%)	0 (0%)	
	D	14 (31.8%)	10 (22.7%)	6 (13.6%)	
	E	26 (59.1%)	33 (75.0%)	37 (84.1%)	
Posterior *n* = 42	C	10 (23.8%)	0 (0%)	0 (0%)	< 0.001
	D	11 (26.2%)	4 (9.5%)	4 (9.5%)	
	E	21 (50.0%)	38 (90.5%)	38 (90.5%)	
Anterior–posterior *n* = 52	A	1 (1.9%)	1 (1.9%)	1 (1.9%)	0.002
	C	6 (11.6%)	0 (0%)	0 (0%)	
	D	15 (28.8%)	11 (21.2%)	5 (9.6%)	
	E	30 (57.7%)	40 (76.9%)	46 (88.5%)

**Table 4:** Clinical cure rate of three surgical approaches (%)

Clinical cure rate	Pre-op	Post-op	Last	*P*
Bad	1 (2.0%)	0 (0%)	1 (2.0%)	0.834
Ordinary	1 (2.0%)	0 (0%)	0 (0%)	
Good	20 (45.5%)	17 (40.5%)	13 (25.0%)	
Outstanding	22 (50.5%)	25 (59.5%)	38 (73.1%)	

The incidence of surgical complications in the three groups was significantly different (*P* < 0.05), and the incidence of anterior complications was the lowest (4.55%), compared with the posterior approach (23.81%) and the anterior–posterior combined approach (21.15%) (Table 5). Besides, the preoperative and postoperative ESR and CRP of three different surgical approaches were also analyzed. The results indicated that the ESR in the posterior group (44.46%) showed worse improvement than that in the anterior group (27.86%) and anterior–posterior group (28.29%) (*χ*^2^ = 16.065, *P* < 0.05). The CRP of the patients in the anterior–posterior group achieved better improvement than the other two groups (42.29% vs 51.55% and 51.71%, *χ*^2^ = 7.062, *P* < 0.05) (Table 6).

**Table 5:** Complications of three surgical approaches (%)

Approach	*n*	No. of complications	Rate	*P*
Anterior	44	2	4.55%	0.031
Posterior	42	10	23.81%	
Anterior–Posterior	52	11	21.15%

**Table 6:** Comparison of ESR and CRP of three surgical approaches before and after surgery

Approach	ESR change (mm/h)	Improvement ratio (%)	Mean rank	CRP change (mg/L)	Improvement ratio (%)	Mean rank
Anterior	14.61 ± 9.88	27.86	60.16	11.19 ± 6.71	51.55	79.92
Posterior	23.52 ± 15.19	44.46	90.11	11.57 ± 10.31	51.71	72.12
Anterior–Posterior	14.90 ± 10.31	28.29	60.76	8.42 ± 8.07	42.29	58.57
*χ*^2^			16.065			7.062
*P*			0.000			0.029

Among 138 patients, 23 complications occurred. Two anterior complications were sinus formation, which recovered after a dressing change. Eight in 10 posterior complications were sinus tract formation and wound exudation, 1 underwent debridement and suturing, and the other 7 cases were cured by dressing change. Two in 10 posterior complications were internal fixation loosening, which did not affect stability, and was not treated. In 11 patients who received anterior–posterior combined surgery, 8 cases occurred wound drainage. One case with a pleural burst was cured by repairing, and 1 case with lumbar artery injury had no effect after hemostasis and artery ligation. Besides, a 63-year-old male, with lower extremity deep vein thrombosis, undertook thrombolysis after placing an inferior vena cava filter due to preoperative Frankel C class, lower limb muscle disorders, poor postoperative lower limb exercise, long-term bedridden condition, right lower limb swelling one week after the operation and thrombus formation in the popliteal vein of right lower limb (complete filling type) detected by ultrasound examination. Then, the edema of the lower limb subsided and thrombosis was cured.

## Discussion

The results indicate that anterior surgery has the shortest hospitalization time, the shortest operation time and the least bleeding. Three open surgical approaches varied in operation time, intraoperative blood loss and hospital stay, with a statistical difference (*P* < 0.05). In the anterior–posterior approach group, the average length of hospital stay, operative time and intraoperative blood loss was significantly longer, compared with the other two groups, the average hospital stays were 15 days in the anterior–posterior group, and the average of 13.5 days in other groups. The average time for anterior–posterior surgery was about 210 min, while 140 min in the anterior group and 153 min in the posterior group, respectively. In terms of blood loss, anterior–posterior surgeries were the most, 482 ml on average, anterior 295 ml and posterior 385 ml, respectively. Yu et al. reported a case series of 21 patients who were treated by posterior debridement and internal fixation with bone grafting and suggested the advantages of the posterior approach in the amount of bleeding and the operative time, compared with the 27 patients treated by anterior surgery [16]. Hassan K compared the outcomes of thoracolumbar discitis by anterior and posterior approach, and they found that the anterior approach has advantages over the posterior one in operative time and the amount of bleeding [17].

Kush et al. aimed at whether patients with spinal tuberculosis need surgery and proposed the natural history and level of tuberculosis, they divided the disease into five levels due to symptoms combined with neurological deficits, and the patients with a rating greater than level three are supposed to receive surgery [18]. Zhang et al. indicated that surgery could be a necessary way of the treatment of lumbar tuberculosis and suggested a posterior approach instead of an anterior approach [19, 20]. A cohort study including 74 patients with anterior surgery and 83 patients with posterior surgery reported by Ma et al. also supported the posterior approach in curative effect [21, 22]. Nevertheless, Jin et al. suggested that the anterior surgical approach could achieve early spinal reconstruction and stabilization, and the assistance with posterior internal fixation was mainly applicable to younger patients [23]. Besides, Zeng et al. compared three approaches and indicated that the loss of kyphosis correction was the highest in the anterior group and all three approaches could improve kyphosis and nerve function, while the amount of bleeding was higher in the anterior–posterior combined group [24–27]. In this study, all three surgical methods can significantly improve neurological function and kyphosis angle, as well as reduce ESR, CRP, VAS and ODI score, and achieve satisfactory clinical healing due to complete debridement, effective nerve decompression and the use of chemotherapy drug. The characteristics of spinal tuberculosis, including bone destruction, local pus, dead bone formation and local TB granulomatous inflammation, may lead to the focus of infection of tuberculosis in vertebral body. After regular chemotherapy treatment, surgery is essential to focal cleaning and bone graft reconstruction regardless of the surgical approach [28]. The reduction of postoperative ESR, CRP and pain score and the improvement of dysfunction index, the neurological function score as well as nutrition support and anti-TB therapy could result in the cure of TB focus, the firm bone graft fusion, the local stability of the spine and good long-term follow-up [28].

The longer average length of hospital stays and operative time as well as more intraoperative blood loss appeared in the anterior–posterior group. Besides, a high incidence of complications occurs in the anterior–posterior group, compared with the anterior and posterior approach, which may result from the sinus of the wound caused by incomplete cleaning. At present, the incidence of anti-TB drug resistance is increasing, and the combination of drug use due to intraoperative specimens genetic and drug resistance tests is advocated to avoid the use of single or only two chemotherapy drugs. Furthermore, the duration of bed rest was relatively long in TB patients, which may lead to complications including deep vein thrombosis. Hence, thrombolytic anticoagulant therapy and other preventions are necessary.

Rajasekaran et al. indicated that spinal tuberculosis was considered to be the most common cause of severe kyphosis [29]. In our study, the kyphosis correction effect of posterior surgery was better than that of anterior and anterior–posterior combined surgery, and the loss rate of kyphosis correction in the anterior group was higher. The vertebral plate or articular process must be removed in posterior surgery, after complete lesions clear and bone graft, due to the effect of strong function of the pedicle screw and rod, and the kyphosis could be corrected convex. However, anterior surgery can clearly show the lesions, while the strength of the kyphosis correction was limited. As for anterior–posterior combined surgery, after the anterior lesions clear and the bone graft, the posterior approach is only for pedicle screw fixation assistance, and the strength of the posterior approach should not be too large after the anterior bone graft. To avoid the loosening and displacement of the bone graft, the posterior approach has advantages over the anterior–posterior combined and the anterior approach in kyphotic correction. Both the posterior approach and anterior–posterior combined approach have lower and upper pedicle screw fixation across the lesion level, resulting in small space loss after kyphosis correction, while the anterior approach alone is prone to correction loss due to partial absorption or loosening of bone graft, which is consistent with our follow-up results.

We consider that the treatment of thoracic lumbar vertebral tuberculosis including transthoracic or extrapleural approach of thoracic tuberculosis debridement and rib bone graft fusion combined thoracoabdominal approach thoracic lumbar tuberculosis debridement and iliac bone graft fusion, and extraperitoneal lumbar tuberculosis debridement and iliac bone graft fusion have a curative effect in showing lesions, cleaning up the pus and sequestrum, with short operation time, less bleeding, short hospital stays and fewer complications. There is no difference between neurological function recovery and clinical healing rate among the three surgical approaches. Zhang et al. reported that the posterior approach could achieve good results in the debridement and treat tuberculosis of the upper thoracic spine [20]. On the contrary, we suggested that the anterior approach should be preferred, which is suitable for patients with relatively good physical condition, lesions mainly located in the anterior middle column, the vertebral collapse that needs height reconstruction and anterior spinal cord compression. The posterior surgical approach could achieve good effect in the treatment of thoracolumbar tuberculosis, which is suitable for patients with vertebral appendix tuberculosis and posterior spinal cord compression, or those whose damage to the vertebral body was not serious and the pus was less. However, the posterior approach may destroy the structure of the vertebral body, resulting in the nidus to the back. The anterior–posterior combined approach is suitable for more serious cases with spinal instability, and difficult anterior fixation, and the disadvantages of this approach are the large wound, intraoperative position change, long period of anesthesia and increasing rate of surgical complications.

Several severe postoperative complications need to be noticed. ①Pleural burst: In thoracic and thoracolumbar tuberculosis anterior surgery, the adhesion of pleura and vertebral body could be severe accompanied by paravertebral and psoas major abscess. In the separation of pleura or rib removal of small head, it is also easy to cause pleura rupture. Once the pleura is ruptured, the pleura must be sutured, and the nearby tissue or muscle tamponade followed by purse-string suture. If the suture is difficult to repair, a closed thoracic drainage can be placed. ②Lumbar vascular injury: Spine is surrounded by abundant blood supply, and the STB patients perform vessels with low elasticity and easy to rupture and bleed. For cases with complex anatomy and deep operative field, it is necessary to be patient, meticulous and gentle operation. In order to pursue thorough cleaning, it is not necessary to scrape the pus cavity forcibly to prevent deep bleeding. For vascular injury, the common methods are suture, ligation, vascular clamp or compression hemostasis. ③Internal fixation loosening: Fracture occurs in general and pseudarthrosis formation, and application of internal fixation of spinal tuberculosis needs to be careful. The surgeons are supposed to conduct careful preoperative preparation, careful operation plan formulation and pay attention to the biomechanical properties and product performance. Some scholars do not recommend using internal fixation after anterior lesion clearance and suggest that anterior lesion clearance, posterior internal fixation installation. For the diseased vertebrae, only short screws are used to fix the vertebrae, and the screws only pass through the pedicle and do not enter the lesion, so as to reduce the possibility of spreading the lesion. ④Infection: The high-risk factors of postoperative infection include obesity, smoking, diabetes, malnutrition, long operation time, lax aseptic operation, etc. Prophylactic use of antibiotics before operation, continued use for 3–5 days after operation, standardized preoperative and postoperative anti-tuberculosis chemotherapy, thorough cleaning of lesions and abscesses and strengthened nutritional support therapy.

Compared with the other two approaches, the anterior approach has more advantages and lower complications, which should be preferred. The posterior and anterior approaches have many disadvantages and high complications, and the anterior approach cannot be easily replaced.

## Data Availability

The datasets supporting the conclusions of this article are included within the article. Raw data can be requested from the corresponding author.

## References

[1] Yang X, Luo C, Liu L, Song Y, Li T, Zhou Z, Hu B, Zhou Q, Xiu P (2020). Minimally invasive lateral lumbar intervertebral fusion versus traditional anterior approach for localized lumbar tuberculosis: a matched-pair case control study. Spine J Off J North Am Spine Soc.

[2] Cheung W, Luk K (2013) Clinical and radiological outcomes after conservative treatment of TB spondylitis: is the 15 years' follow-up in the MRC study long enough?. Eur Spine J Off Publ Eur Spine Soc Eur Spinal Deform Soc Eur Sec Cerv Spine Res Soc 10.1007/s00586-012-2332-x 10.1007/s00586-012-2332-x PMC3691409 22565800

[3] Konstam P, BLESOVSKY A,  (1962). The ambulant treatment of spinal tuberculosis. Br J Surg.

[4] Darbyshire MWPoTotS Five-year assessment of controlled trials of short-course chemotherapy regimens of 6 9 or 18 months’ duration for spinal tuberculosis in patients ambulatory from the start or undergoing radical surgery. Int Orthop 1999; 23(2): 73 10.1007/s002640050311 PMC3619789 10422019

[5] Smith M (1977). Five year assessment of controlled trials of inpatient and outpatient treatment and of plaster of Paris jacket for tuberculosis of the spine in children on standard chemotherapy : fifth report of the medical research council working party on tuberculosos o. J Pediatr Surg.

[6] Darbyshire J, Fox W, Griffiths MD, Tall MR (1993). Controlled trial of short-course regimens of chemotherapy in the ambulatory treatment of spinal tuberculosis. J Bone Joint Surg.

[7] Smith M A 10-Yr assessment of a controlled trial comparing debridement and anterior spinal fusion in the management of tuberculosis of the spine in patients on standard chemotherapy in Hong Kong. Eighth report of the medical research council working party on tuber. 1983; 18: 0–212 10.1302/0301-620X.64B4.7047536 7047536

[8] Shi J, Yue X, Niu N, Zhao C, Qiu H, Wang Z (2017). Application of a modified thoracoabdominal approach that avoids cutting open the costal portion of diaphragm during anterior thoracolumbar spine surgery. Eur Spine J Off Publ Eur Spine Soc Eur Spinal Deform Soc Eur Sec Cerv Spine Res Soc.

[9] Zhong Y, Yang K, Ye Y, Huang W, Liu W, Luo J (2021). Single posterior approach versus combined anterior and posterior approach in the treatment of spinal tuberculosis: a meta-analysis. World Neurosurg.

[10] Muheremu A, Niu X, Wu Z, Tian W Study on anterior and posterior approaches for spinal tuberculosis: a meta-analysis. Eur J Orthop Surg Traumatol 2015; 25: 69-7610.1007/s00590-014-1508-y 10.1007/s00590-014-1508-y 25047733

[11] Liu J, Wan L, Long X, Huang S, Dai M, Liu Z (2015). Efficacy and safety of posterior versus combined posterior and anterior approach for the treatment of spinal tuberculosis: a meta-analysis. World Neurosurg.

[12] Zhou Y, Li W, Liu J, Gong L, Luo J (2018). Comparison of single posterior debridement, bone grafting and instrumentation with single-stage anterior debridement, bone grafting and posterior instrumentation in the treatment of thoracic and thoracolumbar spinal tuberculosis. BMC Surg.

[13] Yao Y, Zhang H, Liu H, Zhang Z, Tang Y, Zhou Y (2017). Prognostic factors for recovery after anterior debridement/bone grafting and posterior instrumentation for lumbar spinal tuberculosis. World Neurosurg.

[14] Cui X, Li L, Ma Y (2016). Anterior and posterior instrumentation with different debridement and grafting procedures for multi-level contiguous thoracic spinal tuberculosis. Orthop Surg.

[15] Wang X, Pang X, Wu P, Luo C, Shen X (2014). One-stage anterior debridement, bone grafting and posterior instrumentation vs. single posterior debridement, bone grafting, and instrumentation for the treatment of thoracic and lumbar spinal tuberculosis. Eur Spine J Off Publ Eur Spine Soc Eur Spinal Deform Soc Eur Sec Cerv Spine Res Soc.

[16] Yu W, Lou C, Liu F, He D (2016). Clinical efficacy of one stage posterior debridement joint graft fixation for lumbar vertebral fractures in spinal tuberculosis patients with compression. Eur Rev Med Pharmacol Sci.

[17] Hassan K, Elmorshidy E (2016). Anterior versus posterior approach in surgical treatment of tuberculous spondylodiscitis of thoracic and lumbar spine. Eur Spine J Off Publ Eur Spine Soc Eur Spinal Deform Soc Eur Sec Cerv Spine Res Soc.

[18] Kumar K (2016). Spinal tuberculosis, natural history of disease, classifications and principles of management with historical perspective. Eur J Orthop Surg Traumatol Orthop Traumatol.

[19] Zhang H, Guo Q, Guo C, Wu J, Liu J, Gao Q, Wang Y (2017). A medium-term follow-up of adult lumbar tuberculosis treating with 3 surgical approaches. Medicine.

[20] Zhang H, Sheng B, Tang M, Guo C, Liu S, Huang S, Gao Q, Liu J, Wu J (2013). One-stage surgical treatment for upper thoracic spinal tuberculosis by internal fixation, debridement, and combined interbody and posterior fusion via posterior-only approach. Eur Spine J Off Publ Eur Spine Soc Eur Spinal Deform Soc Eur Sec Cerv Spine Res Soc.

[21] Ma Y, Cui X, Li H, Chen X, Cai X, Bai Y (2012). Outcomes of anterior and posterior instrumentation under different surgical procedures for treating thoracic and lumbar spinal tuberculosis in adults. Int Orthop.

[22] Cui X, Ma Y, Chen X, Cai X, Li H, Bai Y (2013). Outcomes of different surgical procedures in the treatment of spinal tuberculosis in adults. Med Princ Pract Int J Kuwait Univ Health Sci Centre.

[23] Jin D, Qu D, Chen J, Zhang H (2004). One-stage anterior interbody autografting and instrumentation in primary surgical management of thoracolumbar spinal tuberculosis. Eur Spine J Off Publ Eur Spine Soc Eur Spinal Deform Soc Eur Sec Cerv Spine Res Soc.

[24] Zeng H, Zhang P, Shen X, Luo C, Xu Z, Zhang Y, Liu Z, Wang X (2015). One-stage posterior-only approach in surgical treatment of single-segment thoracic spinal tuberculosis with neurological deficits in adults: a retrospective study of 34 cases. BMC Musculoskelet Disord.

[25] Zeng H, Shen X, Luo C, Xu Z, Zhang Y, Liu Z, Wang X (2015). Comparison of three surgical approaches for cervicothoracic spinal tuberculosis: a retrospective case-control study. J Orthop Surg Res.

[26] Zeng H, Shen X, Luo C, Xu Z, Zhang Y, Liu Z, Wang X, Cao Y (2016). 360-degree cervical spinal arthrodesis for treatment of pediatric cervical spinal tuberculosis with kyphosis. BMC Musculoskelet Disord.

[27] Zeng H, Zhang Y, Shen X, Luo C, Xu Z, Liu Z, Liu X, Wang X (2015). Staged treatment of thoracic and lumbar spinal tuberculosis with flow injection abscess. Int J Clin Exp Med.

[28] Khanna K, Sabharwal S (2019). Spinal tuberculosis: a comprehensive review for the modern spine surgeon. Spine J Off J North Am Spine Soc.

[29] Rajasekaran S (2012). Kyphotic deformity in spinal tuberculosis and its management. Int Orthop.

